# Supplementary material for: Duplicated ribosomal protein paralogs promote alternative translation and drug resistance
Source: Nat Commun. 2022 Aug 23;13:4938. doi: 10.1038/s41467-022-32717-y (PMC9399092; doi:10.1038/s41467-022-32717-y)
Supplement: Supplementary file 1 — Supplementary Information [file 41467_2022_32717_MOESM1_ESM.pdf]

a

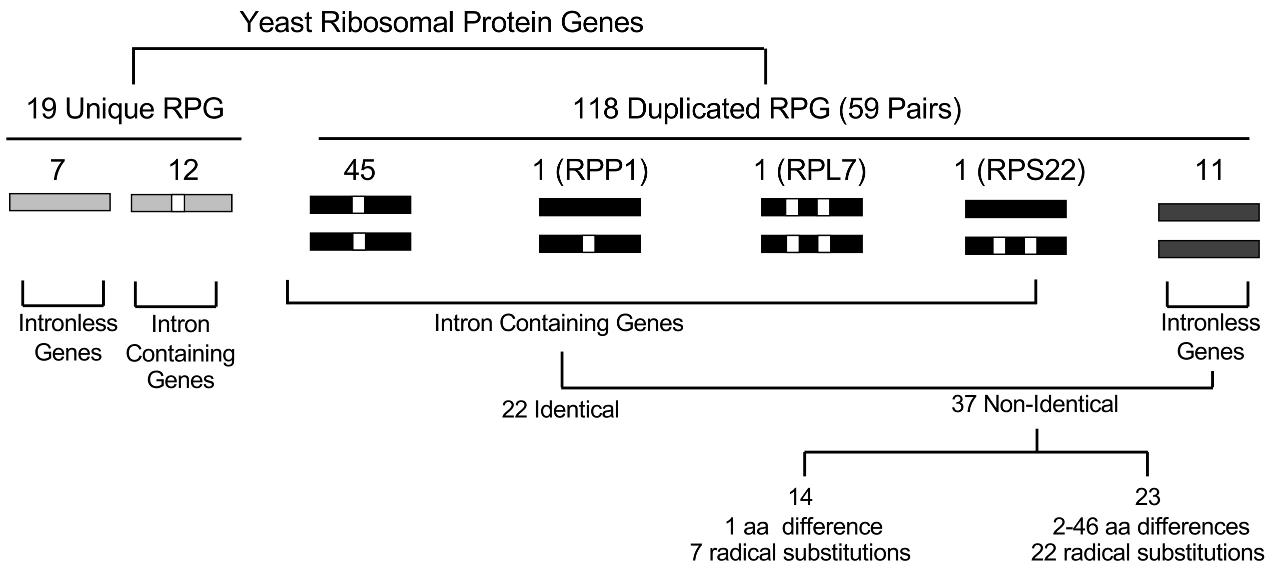

b

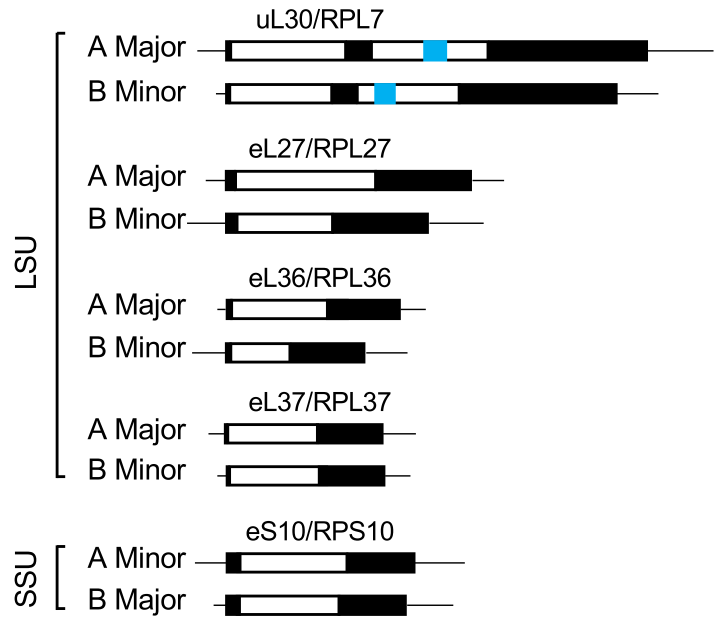

Sequence Identity in Percent

	5' UTR	ORF	Intron	3' UTR
uL30/RPL7	58.5	96.7	60.5	62.1
eL27/RPL27	64.6	89.8	58.7	63.1
eL36/RPL36	63.6	89.4	65.3	67.1
eL37/RPL37	70.0	89.9	56.7	64.8
eS10/RPS10	68.3	93.1	59.0	63.1

Supplementary Fig. 1. Classification and characteristics of yeast ribosomal protein genes. **a,** Schematic representation of yeast RPGs. RPGs were separated based on gene duplication, number of introns and number of amino acid difference between ohnologs. Non-identical paralogs contain at least one amino acid difference. Radical substitution indicates change in the amino acid that may cause charge or structural change. Black boxes indicate exons and white boxes indicate introns. **b,** Characteristics of the RPGs used to generate homogenized yeast strains. Genes coding for large (LSU) and small ribosomal subunits (SSU) proteins are indicated on the left. Both classical and universal protein names are indicated on the top of each gene pairs. Black, blue and white boxes indicates, exon, snoRNA and introns, respectively. The percent sequence identity between the different regions of each pair of genes is indicated on the right.

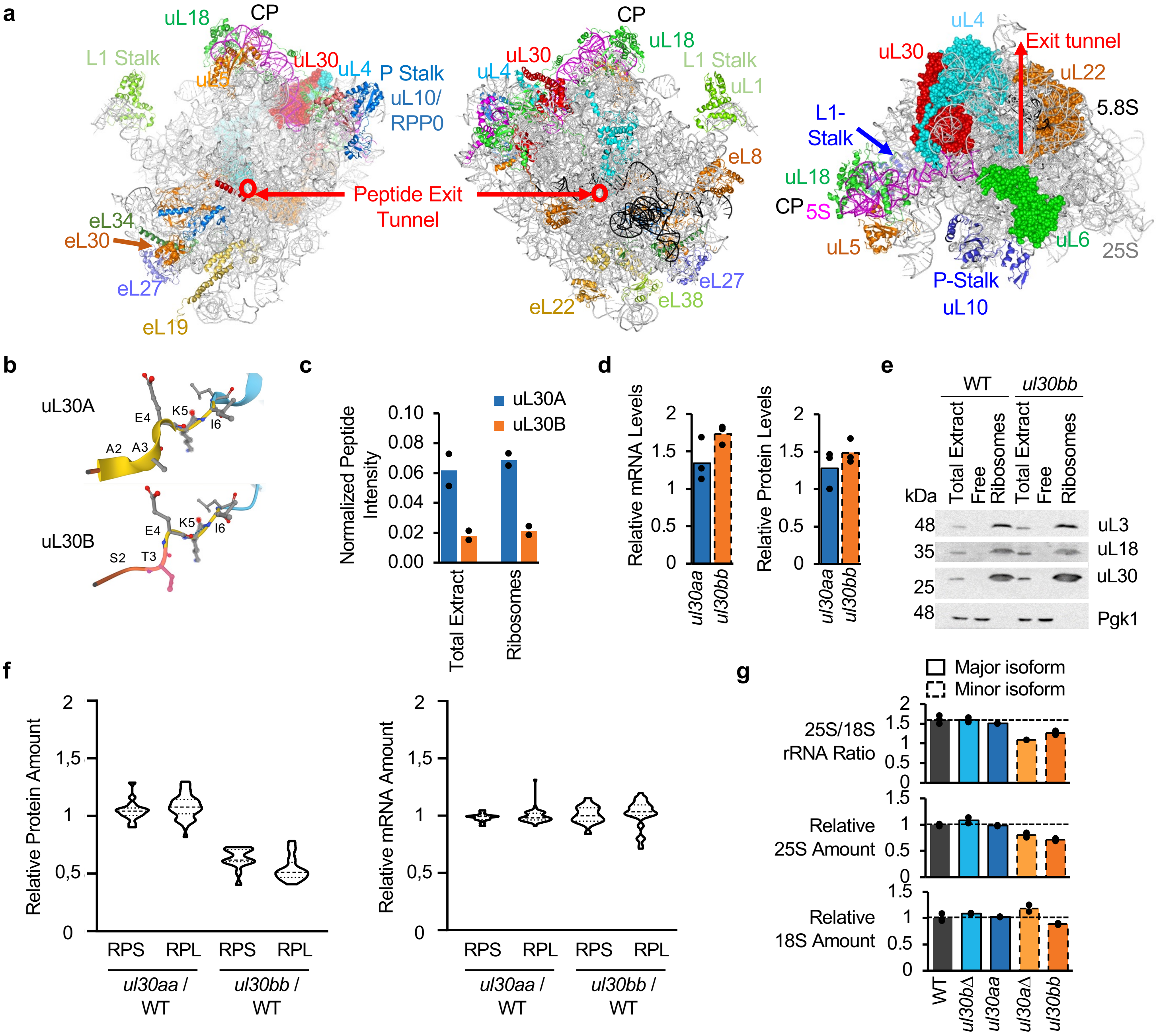

Supplementary Fig. 2. Impact of *uL30/RPL7* paralog on protein and ribosome production. **a,** Position of uL30/RPL7 protein within the 60S subunit. The ribosome structure was obtained from PDB file 5JUO (https://www.rcsb.org/structure/5JUO) originating from Ben-Shem et al., 2011, Science 334:1524. uL30 is indicated in red spheres. The rRNA and spatially or functionally related proteins are labeled. CP and P-stalk indicate the central protuberance and P-stalk base. **b,** A N-terminal ribbon structure is specific to uL30A as predicted by secondary structure comparison of the N-termini of uL30 paralogs from AlphaFold Protein Structure Database. **c,** Paralog-specific peptides were detected and normalized to 60S RPs using Swath MRM in total extract and ribosomes containing fractions from sucrose sedimentation in = 2 biologically independent samples. **d,** Total uL30 mRNA was detected in *ul30aa* or *ul30bb* cells using common primers in qRT-PCR and shown relative to wild-type (left panel). The amount of uL30 protein in *ul30aa* or *ul30bb* cells was determined as in (**c**) and shown relative to WT (right panel). The data is from n = 3 biologically independent samples. **e,** Western blot of uL30, uL3, uL18 and the control Pgk1 proteins from total extracts and sucrose sedimentation fractions. An example is shown from n = 2 biologically independent experiments. Pgk1 and uL3 have similar molecular weights and were run on separate gels with repeats of uL30 and uL18. **f,** Violin plots showing the change in protein (left panel) and mRNA (right panel) produced by different RPGs in homogenized strains compared to WT. Peptides common to both paralogs were used for dRPGs protein quantification and TPMs from both paralogs were added for mRNA assessment. The dotted lines represent the first and third quartiles, the dashed lines represent the median. 16 RPS and 40 RPL were quantified in both dataset and presented in the plots. Proteomic data comes from n = 3 and Rnomic data comes from n = 2 biologically independent samples. **g,** rRNA quantification using capillary electrophoresis. The data comes from n = 3 WT and n = 2 biologically independent samples for the mutant strains.

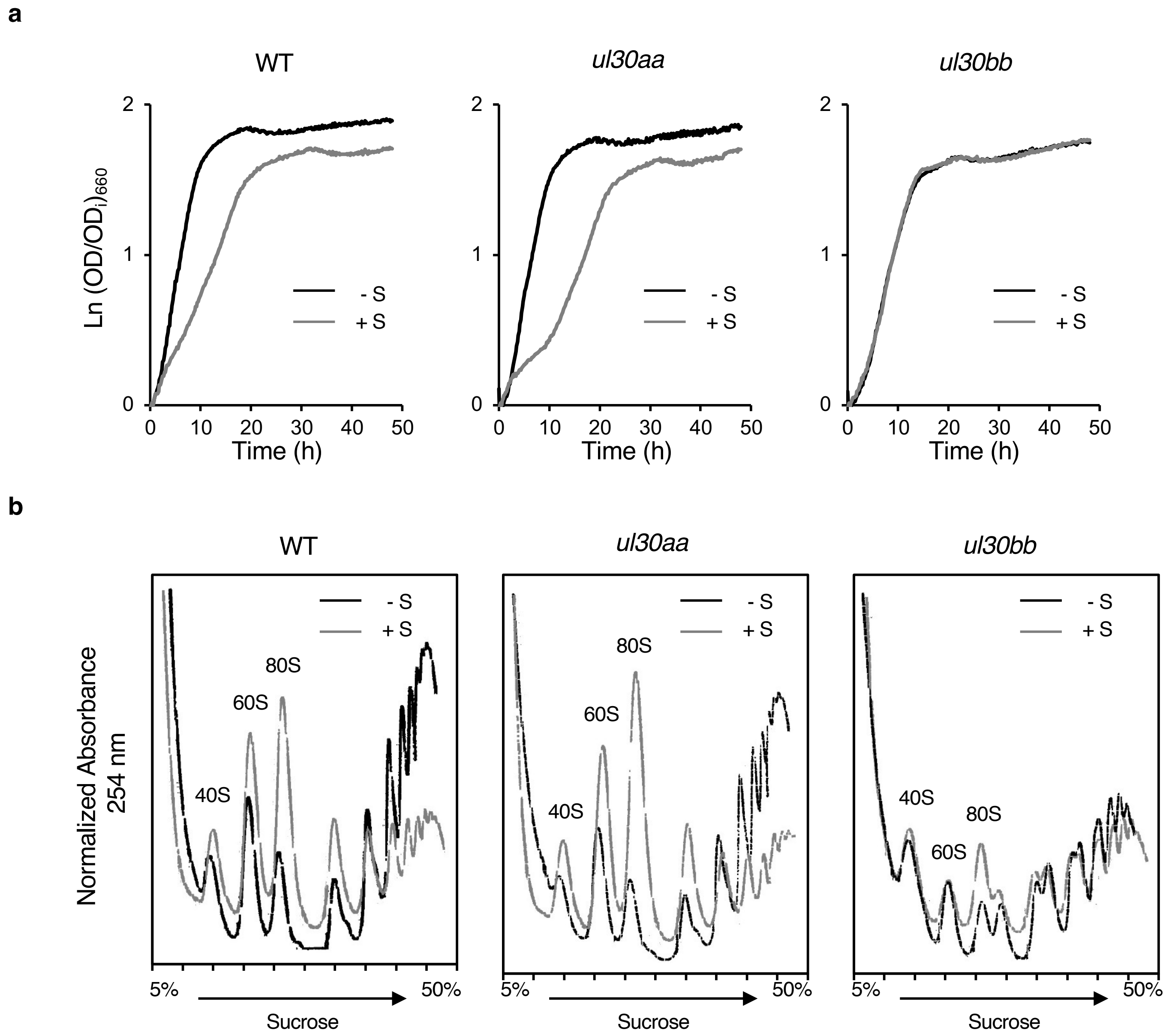

Supplementary Fig. 3. Features and impact of uL30/RPL7 paralogs on cell growth and translation. **a,** Comparison of the growth profile of wildtype (WT) and homogenized (*ul30aa* and *ul30bb*) strains in the presence (+ S) or the absence (- S) of staurosporine. **b,** Comparison of the polysome profile generated from wildtype (WT) and homogenized (*ul30aa* and *ul30bb*) strains strains grown in the presence (+ S) or the absence (- S) of staurosporine. The position of the monosomes and subunits is shown on top. Curves shown are representative examples of n = 3 biologically independent samples.

a

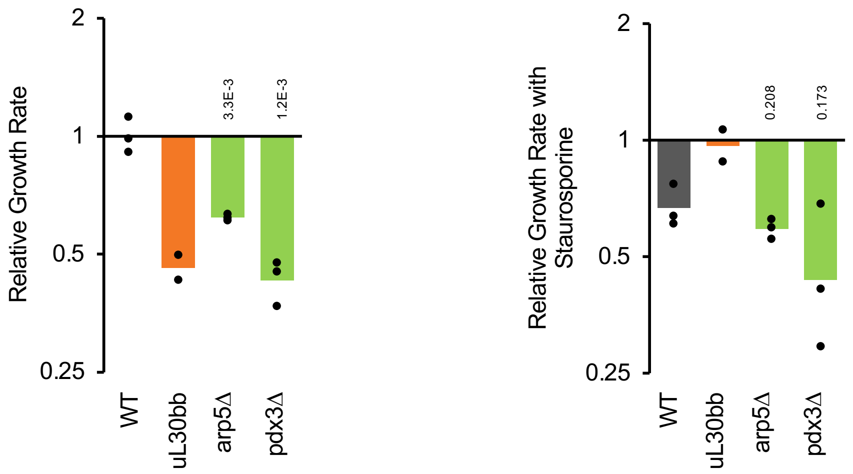

b

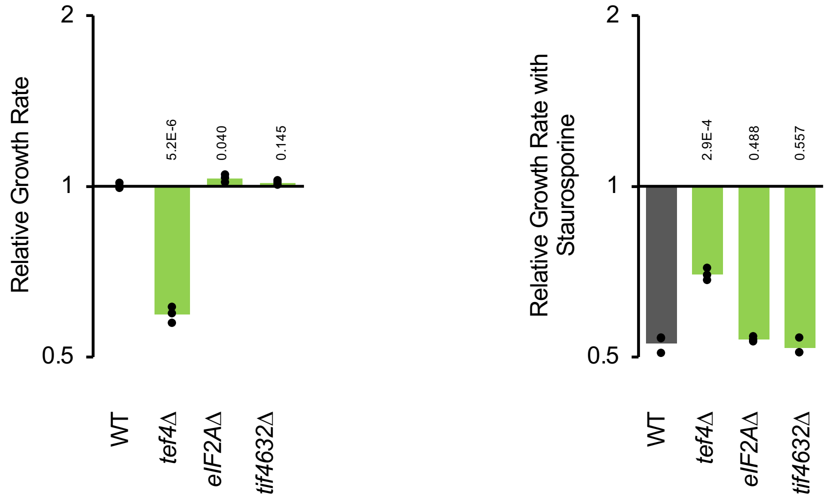

Supplementary Fig. 4. Effect of changes in growth rate and translation efficiency related to cell resistance to staurosporine. **a,** Reduced growth rate is not sufficient for the increased resistance to staurosporine. Relative growth rates of slow growing strains (arp5Δ and pdx3Δ) were compared to those obtained with *ul30bb* and WT strains both in the absence (left panel) and the presence of staurosporine (right panel). **b,** Inhibition of translation elongation and not translation initiation induces resistance to staurosporine. The growth of strains carrying deletion of the translation elongation factor *TEF4,* the initiation factors *eIF2A* and *TIF4632* genes was monitored in the absence (left panel) and the presence (right panel) of staurosporine and the relative growth rates calculated and shown in the form of bar graphs. The data obtained from n = 2 *ul30bb* and n = 3 biologically independent samples for all other strains are indicated by the black circles The significance of difference in growth relative to that obtained with wild type strains is indicated above, when appropriate, as calculated with two-tailed unpaired t-test.

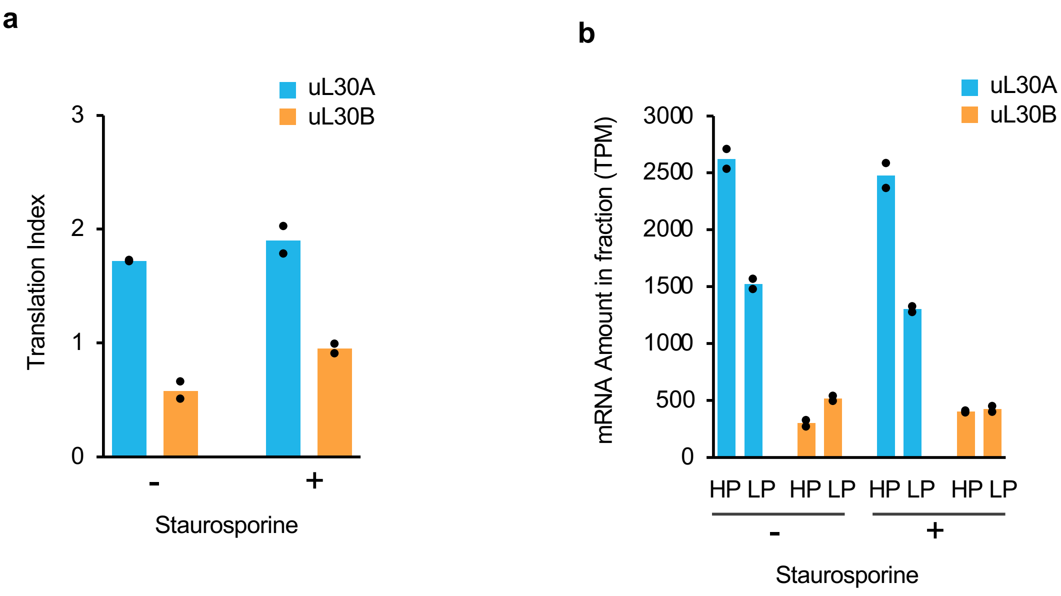

Supplementary Fig. 5. **a**, Translation index of uL30A and uL30B from RNA-seq in WT in untreated and staurosporine treated cells (raw data of Fig. 2e). **b**, TPM values of the uL30A and uL30B mRNAs in heavy (HP) and light (LP) polysome fractions used for the translation index calculation in (**a**). Data points correspond to n = 2 biologically independent samples.

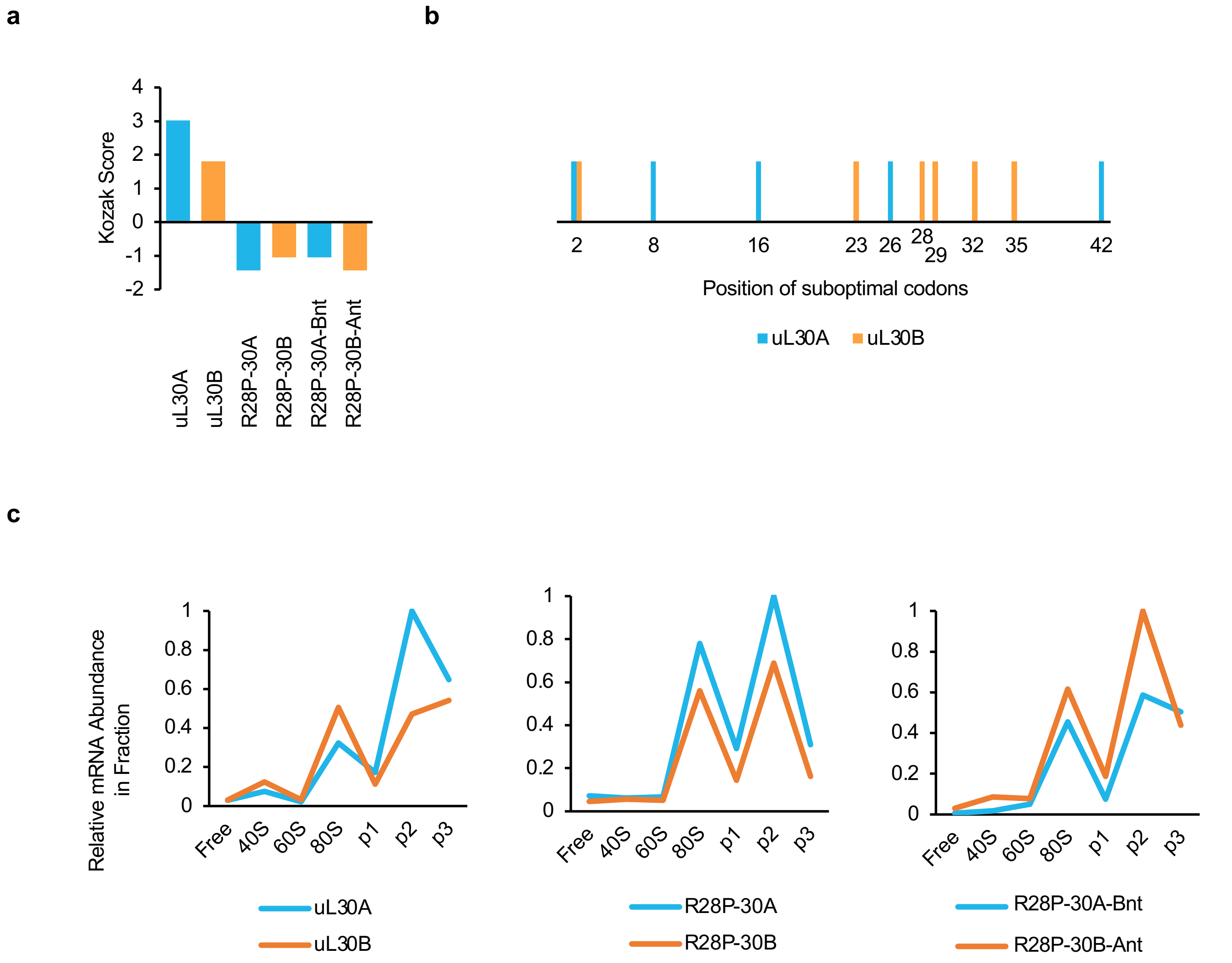

Supplementary Fig. 6. uL30A paralog mRNA is preferentially translated. **a,** The Kozak score (optimal start codon context) was calculated for each construct and presented in the form of a bar graph. **b,** Graph mapping the distribution of suboptimal codons in the first 42 amino acids of uL30A and uL30B. **c,** Translation profiles of uL30A and uL30B in WT and constructs. uL30A and uL30B mRNA from sucrose sedimentation fractions was determined using qRT-PCR. Enrichments were normalized to the highest point per strain pairs and curves represent the average of n = 2 (WT, R28P-30A-Bnt and R28P-30B-Ant) or n = 3 (R28P-30A and R28P-30B) biologically independent samples.

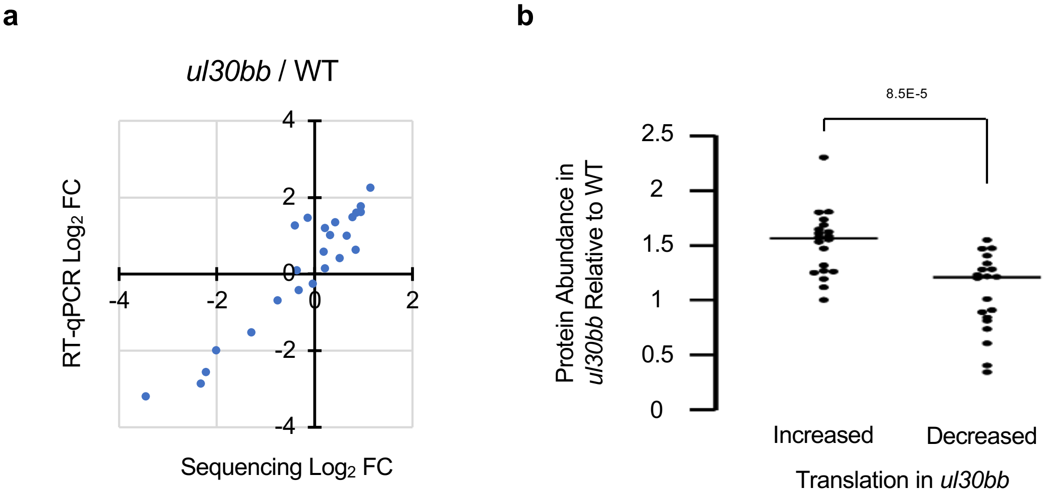

Supplementary Fig. 7. Confirmation of uL30 paralog dependent changes in translation using RT-qPCR and mass spectrometry. **a,** Comparison of the translation index measured by RNA sequence and qRT-PCR. The translation index of 25 genes with altered translation in *ul30bb* strain was calculated using qRT-PCR and compared to that detected by RNA sequencing. The two data set come from n = 2 biologically independent samples and exhibited Pearson correlation coefficient of 0.887. **b,** Protein abundance of the top 20 genes with altered translation in *ul30bb* strain was determined using Swath Multiple Reaction Monitoring (MRM) and compared to WT after normalization to Pgk1p. The data points are average of n = 3 biologically independent samples, the line represent the median. The result of the two-tailed t-test assuming unequal variance is indicated above.

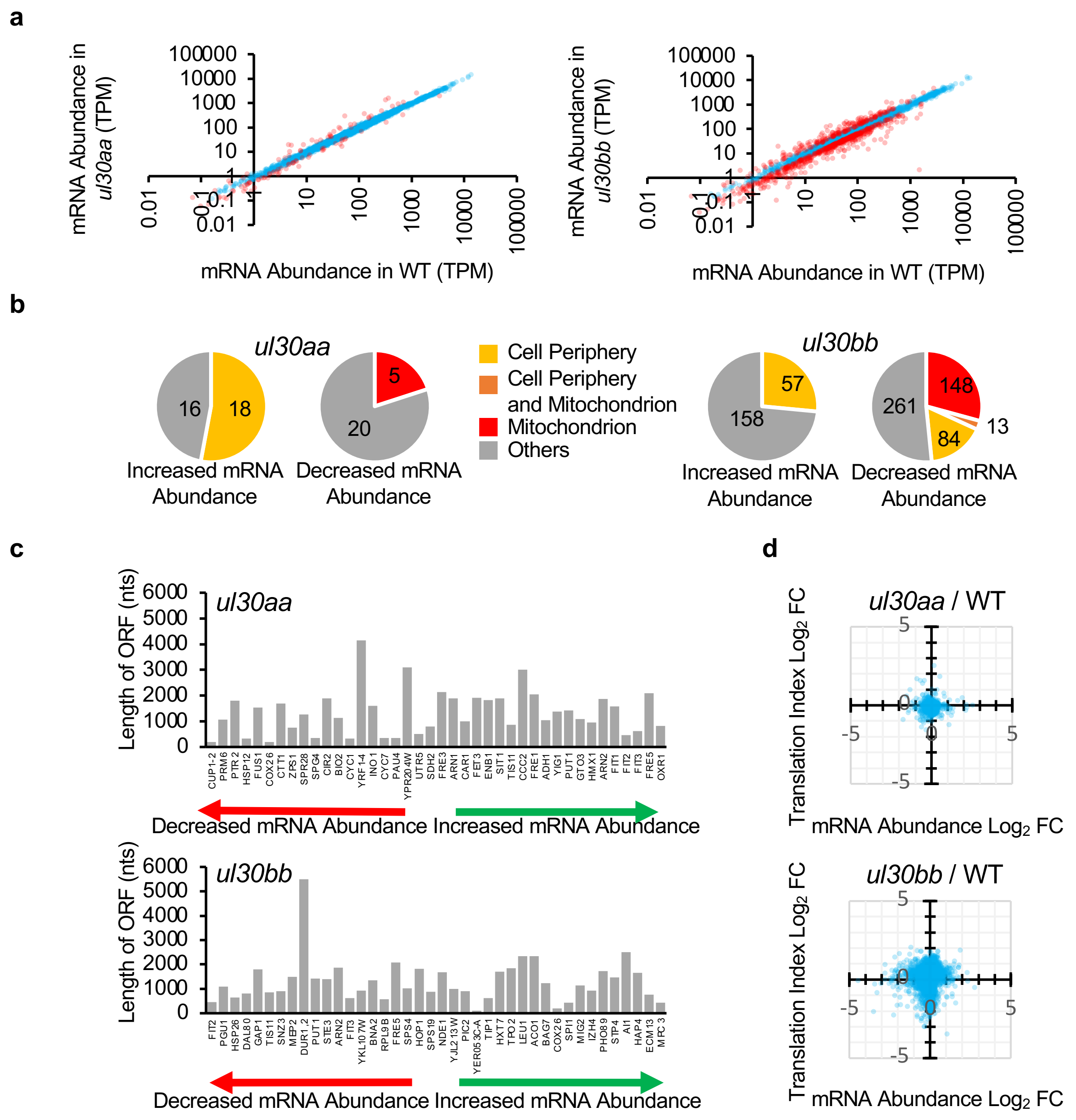

Supplementary Fig. 8. Impact of the homogenization of *uL30/RPL7* paralogs on RNA abundance. **a,** Comparison of expression levels from total RNA sequencing. Genes that vary relative to the wild-type by >Log_2 0.5 are shown in red. Data points are an average of n = 2 biologically independent sample. **b,** Distribution of the number of genes present in enriched component gene ontology categories (p <0.001 with Bonferroni correction) for genes with changes in expression identified in (**a**). An overlap of 13 genes was found in the cell periphery and mitochondrion categories within the mRNAs under-expressed in *ul30bb*. **c,** The top 20 genes over- or under-expressed in *uL30aa* strain (top panel) or *ul30bb* (lower panel) are plotted relative to the length of their ORF in nucleotides. Genes are ordered on the X-axis as function of the magnitude of their change in expression. **d,** Comparison between the change in translation index and mRNA abundance in *ul30aa* and *ul30bb* relative to wild-type strain. The sequencing data comes from n = 2 biologically independent samples. The translation index was calculated described in Fig. 3**a.**

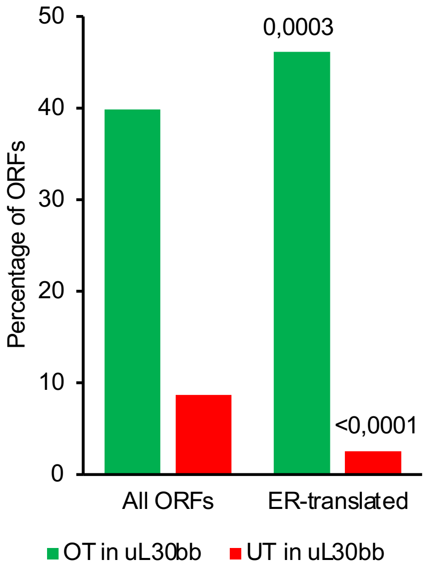

Supplementary Fig. 9. uL30 minor paralog induces the translation of ER-translated genes. The bar graph indicates the percentage of ER-translated ORFs with changed translation index in uL30bb (OT: over translated, UT: under translated). All ORFs: 5252 ORFs quantified in this RNAseq study. ER-translated: 709 ORFs quantified out of 814 mRNAs found enriched in ER bound ribosomes by Medina-Munoz 2020 PNAS (pmid 32907940). Fisher's exact tests were calculated for the number of ORFs found in the over and under translated categories when compared to all ORFs and are related on top of the column

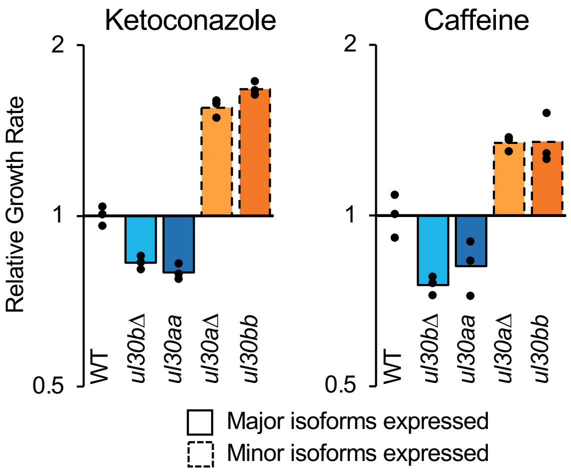

Supplementary Fig. 10. The minor paralog of *uL30/RPL7* increases resistance to drugs inducing cell wall stress. Wild type, *ul30b*Δ, *ul30a*Δ, *ul30aa* and *ul30bb* cells were grown in the presence or the absence of ketoconazole (32 µg/ml, left panel) or caffeine (10 mM, right panel) and the effect on growth rate on the different strains shown relative to that of wild type. The bars represent the average effect observed in n = 3 biologically independent samples shown as data points.

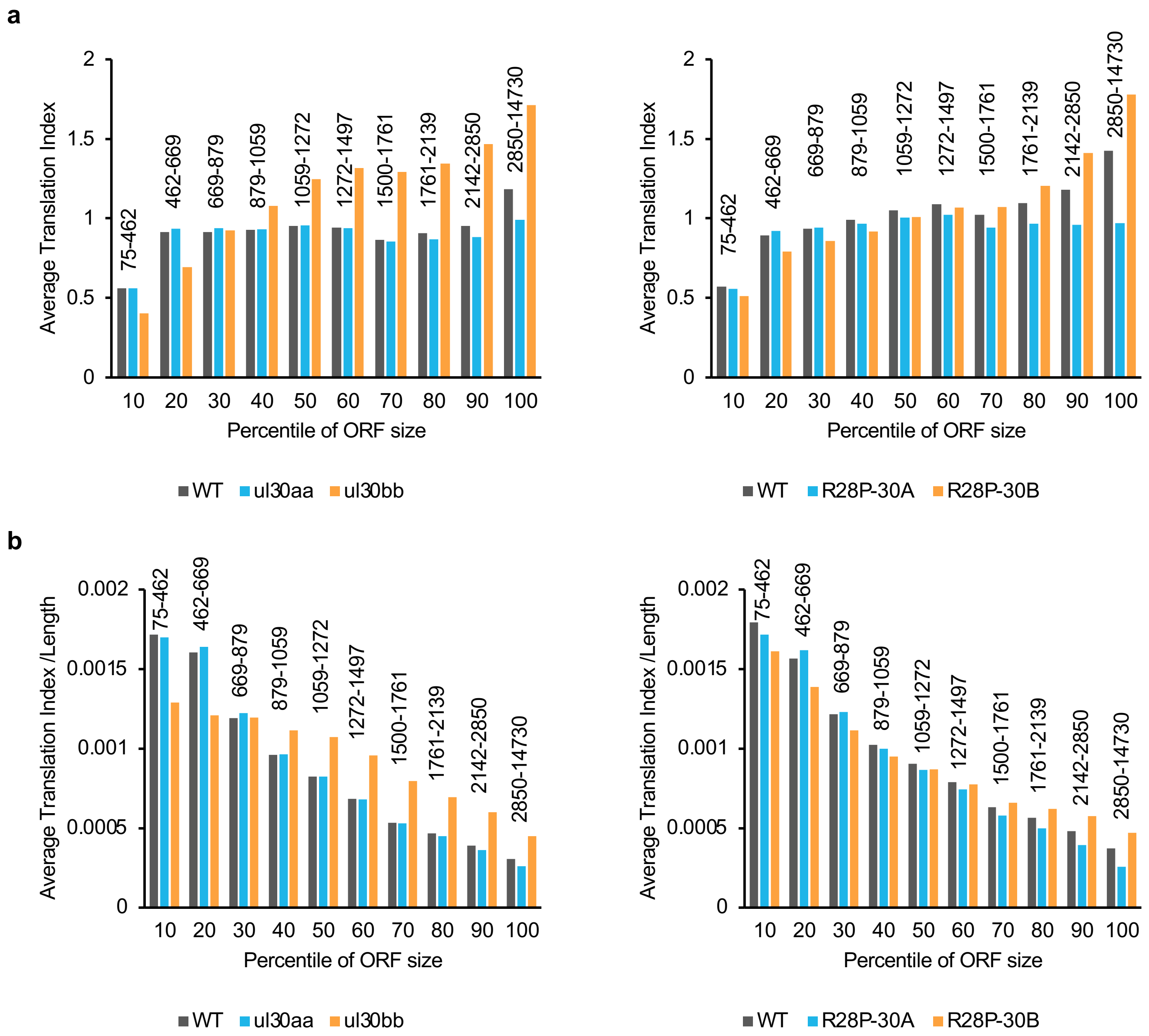

Supplementary Fig. 11. Relationship between the translation index and the length of open reading frames. **a,** Relationship between ORF length and the translation index. Yeast ORFs were separated in 10 bins of 525-526 ORFs according to their size (range in nucleotides noted above bars) and the average translation index (mRNA associated with heavy polyribosome / mRNA associated with light ribosomes, see Figure 3a) was calculated for each bin. The comparison between the values obtained for the wild-type cells, cells expressing two chromosomal copies of *uL30A* (*ul30aa)* and cells expressing two chromosomal copies of *uL30B* (*ul30bb)* is shown on left while the comparison involving cells expressing the plasmid copies of *uL30A* (R28P-30A) or *uL30B* (R28P-30B) genes is indicated on the right panel. **b,** Relationship between ORF length and ribosome density. The translation index was calculated as in (**a**) then divided by ORF's length (number of nucleotides) to calculate ribosome density and the data comparing cells expressing chromosomal copies of uL30 genes (WT, *ul30aa* and *ul30bb*) are shown on the left while those comparing cells expressing uL30 from plasmids (WT, R28P-30A and R28P-30B) are shown on right.

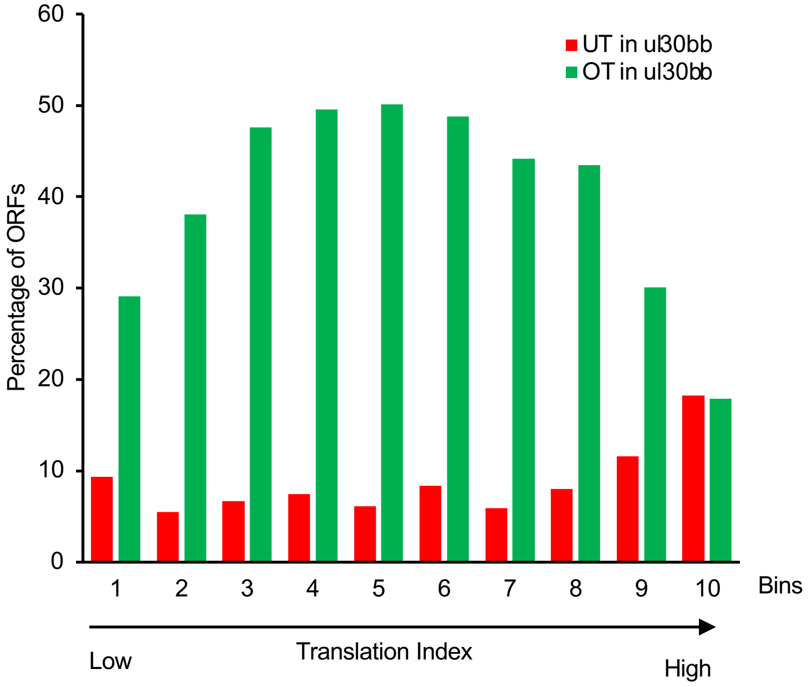

Supplementary Fig. 12. The effect of the minor paralog uL30B on translation is not strictly linked to mRNA level of association with heavy polyribosome. All ORFs were sorted according to their translation index (mRNA associated with heavy polyribosome / mRNA associated with light ribosomes, see Figure 3a) in wild-type cells and separated in 10 bins each containing 525-526 ORFs. Bins were ordered from low (left) to high (right) translation index. The percentage of ORFs in each bin that are found to be under- (UT) or over- (OT) translated in *ul30bb* cells is shown in the form of a bar graph.

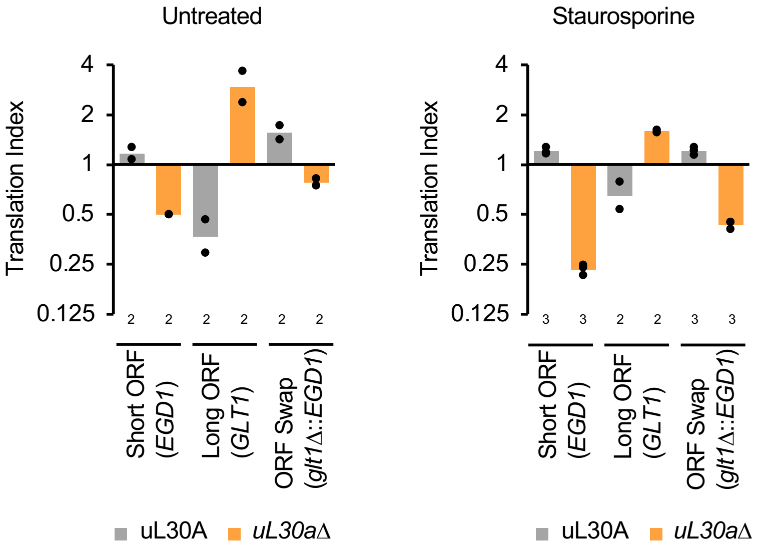

Supplementary Fig. 13. Translation index of short, long and swapped ORF in untreated and staurosporine treated cells expressing uL30A and uL30B (uL30A) or uL30B only (*uL30a*Δ) (raw data of Fig. 4f). Data points correspond to biologically independent samples with n displayed below the graph.

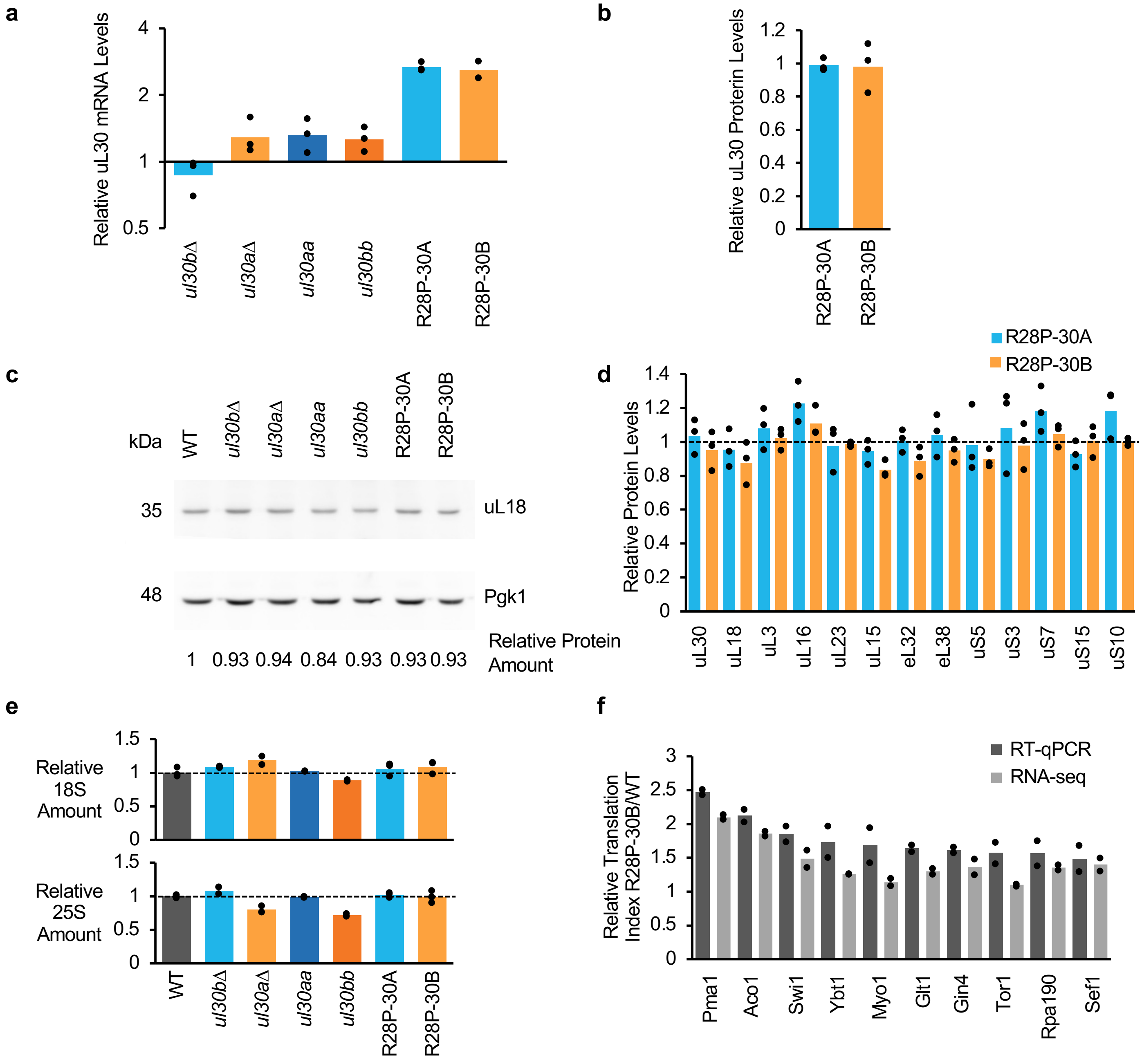

Supplementary Fig. 14. Comparison between RNA, protein and ribosome abundance generated from the chromosomal and plasmid-borne copies of *uL30/RPL7*. **a,** *uL30/RPL7* mRNA level from either plasmid or the chromosomal locus was determined using qRT-PCR and shown relative to the WT for n = 2 R28P-30B and n = 3 biologically independent samples for all other strains. **b,** Quantification of uL30/RPL7 paralogs expressed from plasmids relative to WT after normalization to 60S RPs for n = 3 biologically independent samples using Swath MRM. Both strains express only one version of uL30. **c,** Western blot of uL18 with relative quantification to Pgk1 in strains expressing *uL30/RPL7* paralog from either the chromosomal locus or plasmid calculated from n = 2 biologically independent samples. **d,** Swath MRM was used to quantify RPs normalized to Pgk1 in strains expressing *uL30/RPL7* from plasmids for n = 3 biologically independent samples. **e,** rRNA quantification using capillary electrophoresis for n = 3 (WT, R28P-30A and R28P-30B) or n = 2 biologically independent samples of other strains. **f,** The translation index of 10 long mRNAs (2337-7413 nts) were calculated using qRT-PCR as described in Figure 3**a** and compared to that detected by RNA sequencing. The bar graph represent the average from n = 2 biologically independent samples.

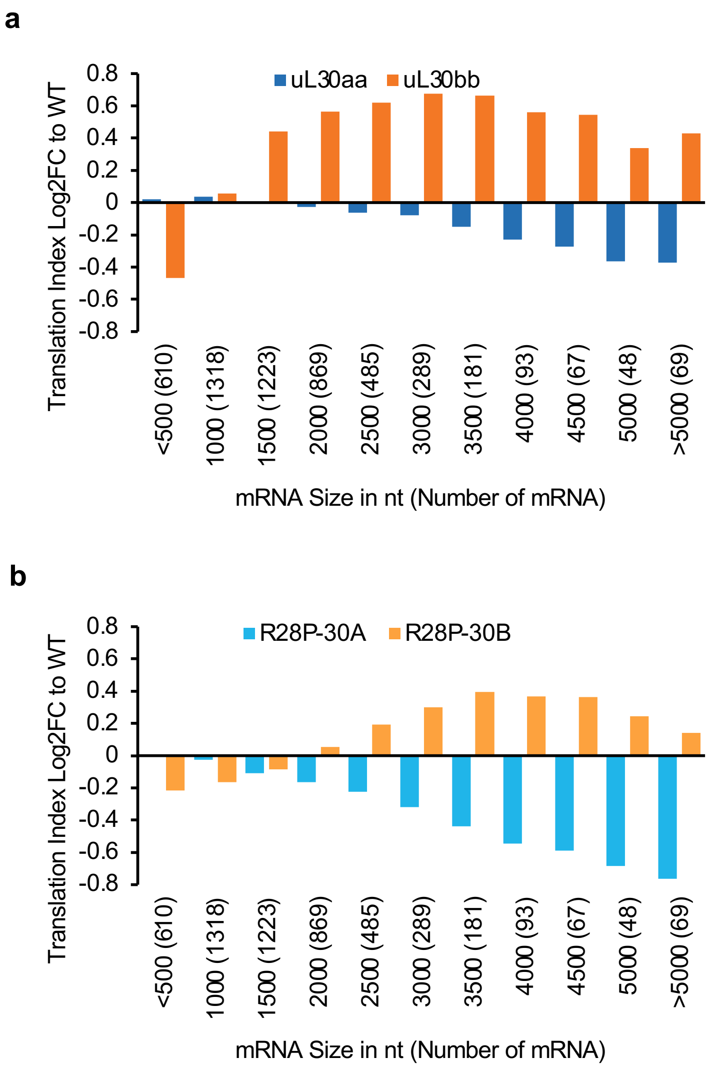

Supplementary Fig. 15. Translation index Log2 fold change to WT for uL30aa and uL30bb (**a**) and R28P-30A and R28P-30B (**b**) according to mRNA size. The average Log2FC for mRNAs in indicated size bins (number of mRNA per bin in parenthesis). The sequencing data comes from n = 2 biologically independent samples (raw data of Fig. 5k).

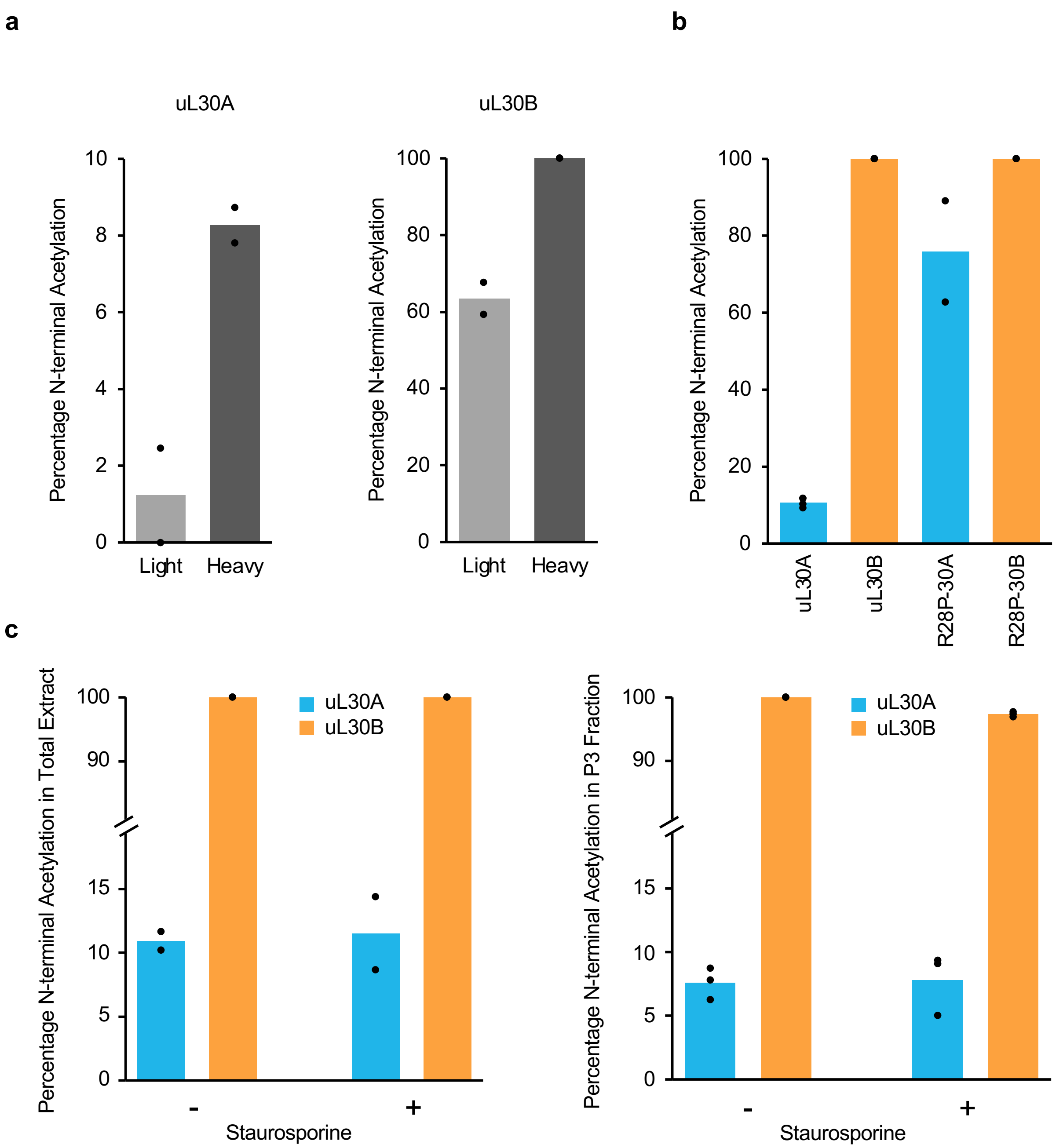

Supplementary Fig. 16. Difference in the acetylation of uL30 proteins associated with different ribosome fractions and growth conditions. **a,** Percent N-terminal acetylation of wild type uL30A (left panel) and uL30B (right panel) proteins associated with the light and heavy polyribosome fractions obtained (raw data of Fig. 6c) for n = 2 biologically independent samples. **b**, Percent N-terminal acetylation of uL30A and uL30B proteins in the total extract of wild type (WT) cells and cells expressing either the A (R28P-30A) or B (R28P-30B) version of the protein from plasmids from n =4 WT and n = 2 R28P-30A and R28P-30B biologically independent samples. **c,** Effect of staurosporine on uL30 acetylation. The percent N-terminal acetylation of uL30A and uL30B was detected in total extract (left panel) and heavy polyribosome (right panel) before and after staurosporine treatment. Points represent data from n = 2 total extract and n = 3 P3 fractions biologically independent samples.

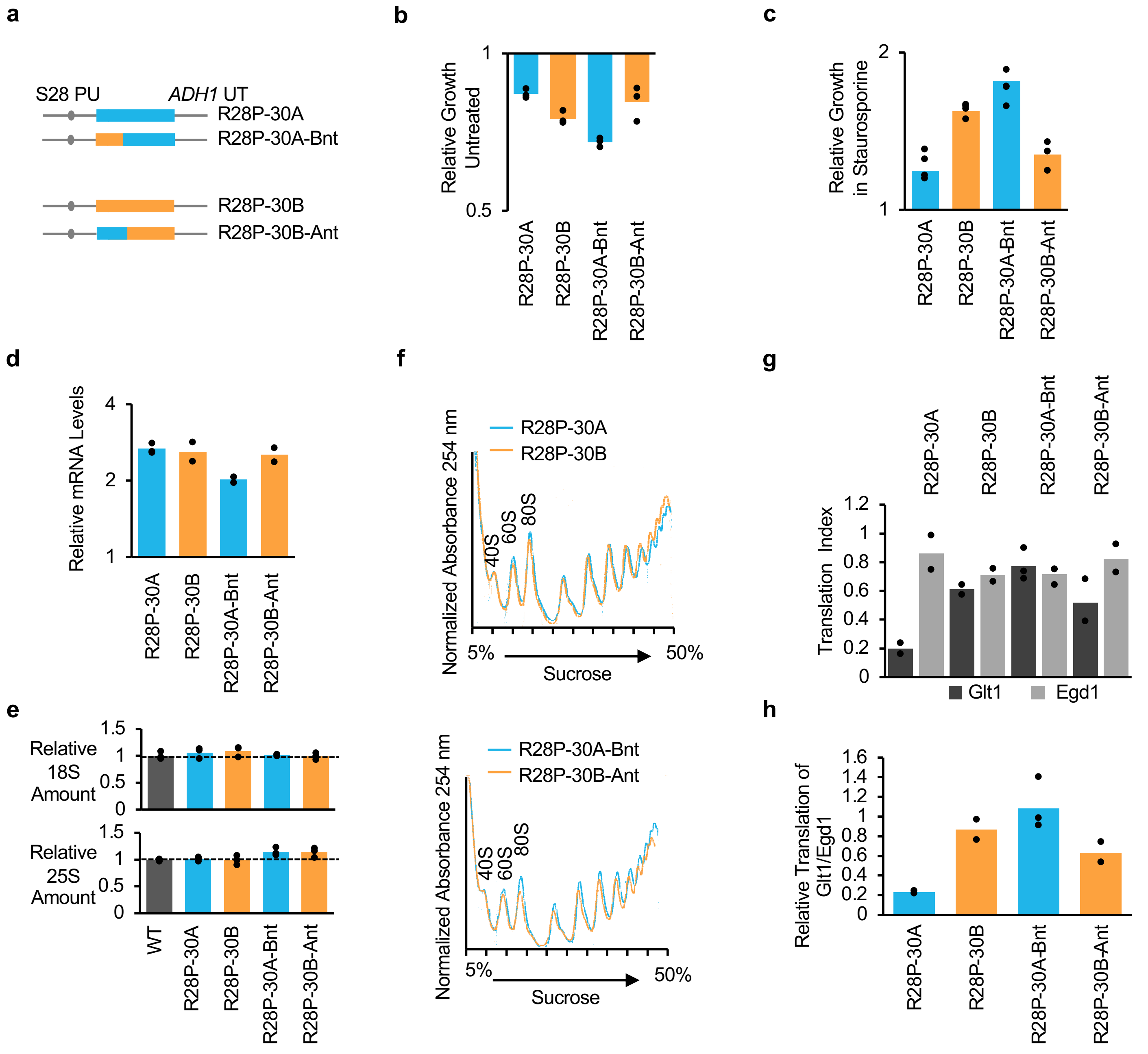

Supplementary Fig. 17. The difference in staurosporine resistance and mRNA size selection is included in the N-terminal part of uL30. **a,** The plasmid-borne uL30 paralogs and mutations used in (**b-h**) are schematically illustrated and the position of the mutations are indicated in the form of boxes. The ORFs of uL30A/RPL7A and uL30B/RPL7B are indicated as light blue and orange boxes. **b,** The relative growth rate of cells expressing one or the other uL30/RPL7 paralog from plasmid or the different mutations was determined in YC complete media and shown in the form of a bar graph with points representing n = 3 biologically independent samples. **c,** The growth rate in the presence of staurosporine was calculated as in (**b**) for strains carrying the different constructs and relative growth effect on each strain compared to wt indicated in the form of a bar graph for n = 3 R28P-30B-Ant and n = 4 biologically independent samples of the other strains. **d,** The mRNA produced from the different constructs was determined using RT-qPCR from n = 3 R28P-30A and n = 2 biologically independent samples of the other strains. **e,** rRNA quantification using capillary electrophoresis and compared to WT for n = 3 biologically independent samples. **f,** Comparison of the polyribosome profiles from cells expressing the different constructs. Representative curves from n = 3 biologically independent samples are shown. **g,** The ratio of mRNA with long (Glt1, 6438 nt) and short (Egd1, 474 nt) open reading frame associated with heavy polyribosome was examined in cells expressing wild-type or mutated versions of uL30/RPL7 paralog using RT-qPCR as explained in Figure 3**a** for n = 3 R28P-30A-Bnt and n = 2 biologically independent samples for all other strains. **h,** Ratio of the translation index of long ORF Glt1 and short ORF Egd1 determined in (**g**). The data points of n = 3 R28P-30A-Bnt and n = 2 biologically independent samples of all other strains are shown.

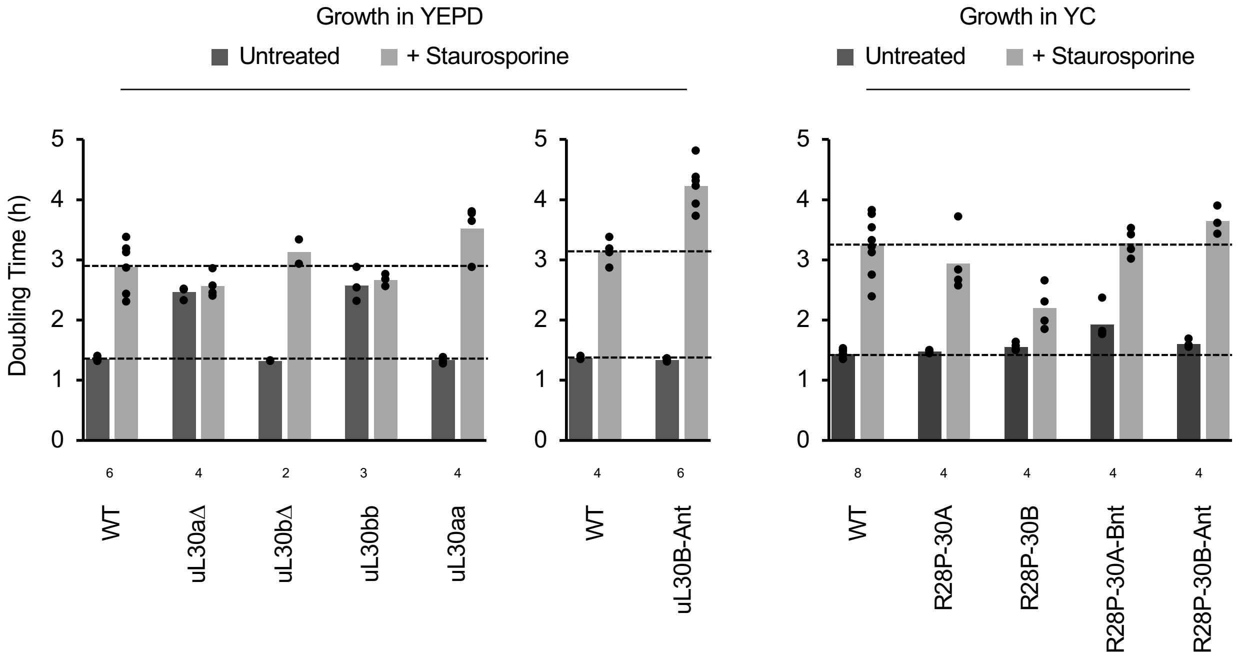

Supplementary Fig. 18. Summary of the effect of different mutations and treatment on cell growth. The doubling time of the different strains grown in either rich (YEPD) or selective (YC) media before or after exposure to staurosporine was calculated and the results shown in the form of bar graph. The data are an average n biologically independent samples as noted below and shown as black dots.

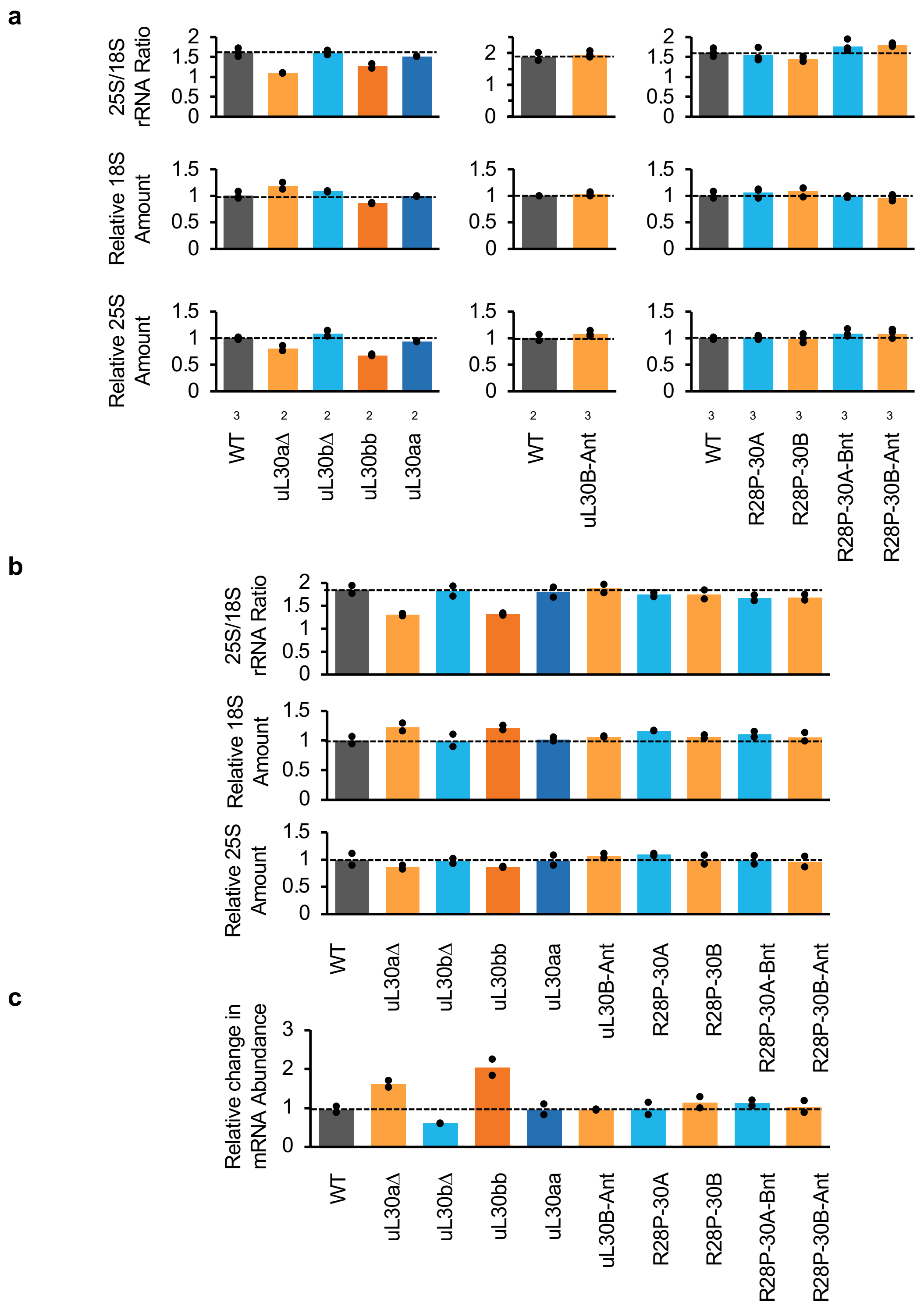

Supplementary Fig. 19. Summary of the effects of mutations and exposure to drugs on the abundance of rRNA and uL30 mRNA. **a,** Effect of uL30 paralog on the abundance of rRNA. The 18S and 25S RNA was extracted from strains expressing different versions of uL30 paralog and rRNA was quantified using capillary electrophoresis. The ratio of the 25S/18S (top panel) as well as the amount of 18S (middle panel) and 25S (bottom panel) rRNA are shown in the form of bar graphs. 18S and 25S values were normalized to wild type (WT) cells. The results n biologically independent samples (noted below) are indicated by the black dots. **b,** Effect of the exposure to staurosporine on the rRNA abundance. The RNA was extracted after 4 hours exposure to staurosporine and the data calculated and presented as in (**a**) for n = 2 biologically independent samples. **c,** Effect of staurosporine on the expression of uL30. The RNA was extracted from strains carrying different version of uL30 genes before or after 4 hours exposure to staurosporine and abundance of uL30 mRNA detected using RT-qPCR. The bar graph showing the ratio of the RNA detected in treated and untreated cells. The results of n = 2 biologically independent samples are indicated by the black dots.
