# Supplementary material for: Duplicated ribosomal protein paralogs promote alternative translation and drug resistance
Source: Nat Commun. 2022 Aug 23;13:4938. doi: 10.1038/s41467-022-32717-y (PMC9399092; doi:10.1038/s41467-022-32717-y)
Supplement: Supplementary file 3 — Description of Additional Supplementary Files [file 41467_2022_32717_MOESM3_ESM.pdf]

Description of Additional Supplementary Files

File Name: Supplementary Data 1
Description: Strains Used in this Study

File Name: Supplementary Data 2
Description: Oligonucleotides Used in this Study

File Name: Supplementary Data 3
Description: Summary of Sequencing Data and Gene Analysis

File Name: Supplementary Data 4
Description: Doubling Times of uL30 Strains

File Name: Supplementary Data 5
Description: Translation Index of WT and homogenized strains
