# Supplementary material for: Duplicated ribosomal protein paralogs promote alternative translation and drug resistance
Source: Nat Commun. 2022 Aug 23;13:4938. doi: 10.1038/s41467-022-32717-y (PMC9399092; doi:10.1038/s41467-022-32717-y)
Supplement: Supplementary file 9 — Reporting Summary [file 41467_2022_32717_MOESM9_ESM.pdf]

nature research

Corresponding author(s): Sherif Abou Elela

Last updated by author(s): Oct 7, 2021

Reporting Summary

Nature Research wishes to improve the reproducibility of the work that we publish. This form provides structure for consistency and transparency in reporting. For further information on Nature Research policies, see our Editorial Policies and the Editorial Policy Checklist.

Statistics

For all statistical analyses, confirm that the following items are present in the figure legend, table legend, main text, or Methods section.

n/a	Confirmed	
☐	☒	The exact sample size (*n*) for each experimental group/condition, given as a discrete number and unit of measurement
☐	☒	A statement on whether measurements were taken from distinct samples or whether the same sample was measured repeatedly
☐	☒	The statistical test(s) used AND whether they are one- or two-sided<br>*Only common tests should be described solely by name; describe more complex techniques in the Methods section.*
☐	☒	A description of all covariates tested
☐	☒	A description of any assumptions or corrections, such as tests of normality and adjustment for multiple comparisons
☐	☒	A full description of the statistical parameters including central tendency (e.g. means) or other basic estimates (e.g. regression coefficient) AND variation (e.g. standard deviation) or associated estimates of uncertainty (e.g. confidence intervals)
☐	☒	For null hypothesis testing, the test statistic (e.g. *F*, *t*, *r*) with confidence intervals, effect sizes, degrees of freedom and *P* value noted<br>*Give P values as exact values whenever suitable.*
☒	☐	For Bayesian analysis, information on the choice of priors and Markov chain Monte Carlo settings
☒	☐	For hierarchical and complex designs, identification of the appropriate level for tests and full reporting of outcomes
☐	☒	Estimates of effect sizes (e.g. Cohen's *d*, Pearson's *r*), indicating how they were calculated

Our web collection on statistics for biologists contains articles on many of the points above.

Software and code

Policy information about availability of computer code

Data collection	Growth curve data was collected using Gen5 v.03.08.01<br>25S and 18S rRNA was quantified using Agilent 2200 TapeStation Controller v.A.02.02<br>qRT-PCR data was acquired using Bio-Rad CFX Maestro 2.0 v.5.0.021.0616<br>ddPCR data was acquired using Bio-Rad Quantasoft v.1.7.4.0917<br>Western blots chemiluminescence was imaged using ImageQuant LAS 4000 v.1.2
Data analysis	Microsoft Excel 365 v.2108 and GraphPad Prism v.8.3.0 were used for general data analysis<br>25S/18S ratios were determined using Agilent TapeStation Analysis Software v.A.02.02<br>ddPCR was quantified using Bio-Rad Quantasoft Analysis Pro v.1.0.5.96<br>Western blots were quantified using Bio-Rad Quantity One v.4.6.9<br>RNA sequencing data has been analyzed with STAR Aligner v.2.5 and Rsubread v.1.16.1<br>Proteomic data has been analyzed with PeakView v.2.2 , Skyline v.4.2 and MaxQuant v.2.0.1.0

For manuscripts utilizing custom algorithms or software that are central to the research but not yet described in published literature, software must be made available to editors and reviewers. We strongly encourage code deposition in a community repository (e.g. GitHub). See the Nature Research guidelines for submitting code & software for further information.

Data

Policy information about availability of data

All manuscripts must include a data availability statement. This statement should provide the following information, where applicable:

- Accession codes, unique identifiers, or web links for publicly available datasets
- A list of figures that have associated raw data
- A description of any restrictions on data availability

Additional RNA-seq data generated in this study have been submitted to the NCBI Gene Expression Omnibus (GEO; https://www. ncbi.nlm.nih.gov/geo) under the accession number GSE133457 and xxx. Proteomic data presented in this study has been submitted to the Peptide Atlas (http://www.peptideatlas.org/) under accession number PASS01404 and xxx. All strains are available upon request. Source data are provided with this paper. All other data supporting the findings of this study are available from the corresponding author on reasonable request.

Field-specific reporting

Please select the one below that is the best fit for your research. If you are not sure, read the appropriate sections before making your selection.

[x] Life sciences [ ] Behavioural & social sciences [ ] Ecological, evolutionary & environmental sciences

For a reference copy of the document with all sections, see nature.com/documents/nr-reporting-summary-flat.pdf

Life sciences study design

All studies must disclose on these points even when the disclosure is negative.

Sample size	Sample size was not determined using statistical methods. At least 2 biological repeats were used for all experiments. Sample size was chosen based on previous experiments and reliability of the measures.
Data exclusions	In RT-qPCR, technical replicates diverging by more than 1.5 SD were removed from the analysis. In mass spectrometry analysis, samples were rejected when target peptide could not be detected.
Replication	All data has been replicated in at least 2 independent biological samples as stated in the text. Special care has to be taken in growth assays from strains expressing uL30 on plasmids or mutants of uL30. Experiments have to be carried from freshly created strains (either from plasmid shuffle or spore dissection) to be reproducible.
Randomization	No sample randomization was done other than picking random colonies on the petri dishes to inoculate the cultures.
Blinding	No blinding was done. All of the analysis was based on unbiased measurements.

Reporting for specific materials, systems and methods

We require information from authors about some types of materials, experimental systems and methods used in many studies. Here, indicate whether each material, system or method listed is relevant to your study. If you are not sure if a list item applies to your research, read the appropriate section before selecting a response.

Materials & experimental systems

n/a	Involved in the study	
[ ]	[x]	Antibodies
[x]	[ ]	Eukaryotic cell lines
[x]	[ ]	Palaeontology and archaeology
[x]	[ ]	Animals and other organisms
[x]	[ ]	Human research participants
[x]	[ ]	Clinical data
[x]	[ ]	Dual use research of concern

Methods

n/a	Involved in the study	
[x]	[ ]	ChIP-seq
[x]	[ ]	Flow cytometry
[x]	[ ]	MRI-based neuroimaging

Antibodies

Antibodies used	Mouse anti-L3, from Warner lab, 1:5000<br>Rabbit anti-L5, from Woolford lab, 1:2000<br>Rabbit anti-L7, #A300-741A-M Bethyl Laboratories, Lot# A300-741A-M-3, 1:1000<br>Mouse anti-Pgk1, #459250 Invitrogen-Thermo Fisher, Lot#F1284, 1:10,000<br>Donkey anti-rabbit IgG-HRP, #NA934V GE Healthcare, Lot#16953209, 1:5000<br>Goat anti-mouse IgG-HRP, #NA931V GE Healthcare, Lot#172212127, 1:5000

Validation

Mouse anti-L3 and Rabbit anti-L5 antibodies have been used to detect yeast ribosomal proteins L3 and L5 respectively in several publications including Vilardell and Warner (1997) Mol. Cell. Biol.; Deshmukh et al. (1995) J. Biol. Chem; Rosado et al. (2007) Nucleic Acids Res. Bands of the expected size (44 and 34 kDa) were observed on blots.

Rabbit anti-L7 antibody made against the C-terminus of human ribosomal protein L7 has been successfully used to detect yeast L7 as in Palumbo et al. (2017) G3 Genes. Bands of expected size (28 kDa) were observed on blots.

Mouse anti-Pgk1 has been used as loading control in several articles including Petibon et al. (2016) Nucleic Acids Res. and Parenteau et al. (2019) Nature. Bands of expected size (45 kDa) were observed on blots.
